# Severe stenosis of major intracranial arteries: an important risk factor for infarction complications after combined revascularization in adult patients with ischemic moyamoya disease

**DOI:** 10.1186/s41016-026-00430-0

**Published:** 2026-04-14

**Authors:** Cunxin Tan, Xun Ye, Hongchuan Niu, Ran Duan, Guangchao Shi, Yuanli Zhao, Rong Wang

**Affiliations:** 1 Department of Neurosurgery, Peking University International Hospital, Beijing, China; 2 Department of Neurosurgery, Beijing Tiantan Hospital, Capital Medical University, Beijing, China; 3 Department of Neurosurgery, Peking Union Medical College Hospital, Beijing, China

**Keywords:** Moyamoya disease, Postoperative cerebral infarction, Revascularization

## Abstract

**Background:**

Revascularization is the main effective treatment for moyamoya disease Moyamoya disease (MMD) but is associated with a high risk of postoperative cerebral infarction. We aimed to analyze the correlation between lesions in intracranial major arteries and postoperative cerebral infarction.

**Methods:**

Adult patients with ischemic MMD were enrolled in this analysis from June 1, 2021, to October 31, 2024. The preoperative clinical characteristics and features of digital subtraction angiography were recorded. Postoperative computed tomography or magnetic resonance imaging was performed to identify infarction complications. The Suzuki stage and lesions of the intracranial major arteries were analyzed to determine their correlations with postoperative cerebral infarction.

**Results:**

A total of 119 adult patients with ischemic moyamoya disease were included in the final analysis. When the degree of stenosis was considered a disordered variable and the normal artery was taken as the reference, the logistic regression analysis showed that severe stenosis of the internal carotid artery (ICA), anterior cerebral artery (ACA), middle cerebral artery (MCA), and posterior cerebral artery (PCA) was significantly correlated with postoperative infarction (ICA (OR, 8.833; 95% CI, 0.730–106.821; *p* = 0.046), ACA (OR, 49.500; 95% CI, 5.083–482.011; *p* = 0.001), MCA (OR, 24.000; 95% CI, 1.459 − 394.881; *p* = 0.026), and PCA (OR, 54.333; 95% CI, 4.307–685.462;* p* = 0.002)). Digital subtraction angiography (DSA) was performed on three patients with postoperative cerebral infarction and showed acute occlusion or aggravated stenosis of the major intracranial arteries with severe preoperative stenosis.

**Conclusions:**

Severe stenosis of major intracranial arteries is an important risk factor for cerebral infarction after combined revascularization in adult patients with ischemic MMD.

**Supplementary Information:**

The online version contains supplementary material available at 10.1186/s41016-026-00430-0.

## Background

Moyamoya disease (MMD) is a unique cerebrovascular disorder characterized by progressive spontaneous stenosis or occlusion of the distal internal carotid artery (ICA) and the proximal part of its major branches, accompanied by net-like vessels forming at the base of the brain [1–4]. Revascularization is the main effective treatment option [5, 6]. However, the incidence of cerebral ischemia events following surgery is relatively high, approximately 5%, and no satisfactory methods are available to predict these events. Previous studies have reported that risk factors might include a high Suzuki stage, preoperative ischemic presentation, surgery in the acute stage of cerebral infarction, high partial pressure of carbon dioxide (CO_2_), low circulation volume, low hematocrit level, and abnormal blood pressure [7–13]. In the past 3 years, despite efforts to pay attention to the risk factors mentioned in the literature, the incidence of postoperative ischemia has not improved significantly in our center. In our clinical practice, we found that focal severe stenosis of the intracranial major arteries might be a risk factor for severe infarction after surgery. This study aims to study and analyze this issue.

## Methods

**Fig. 1:**
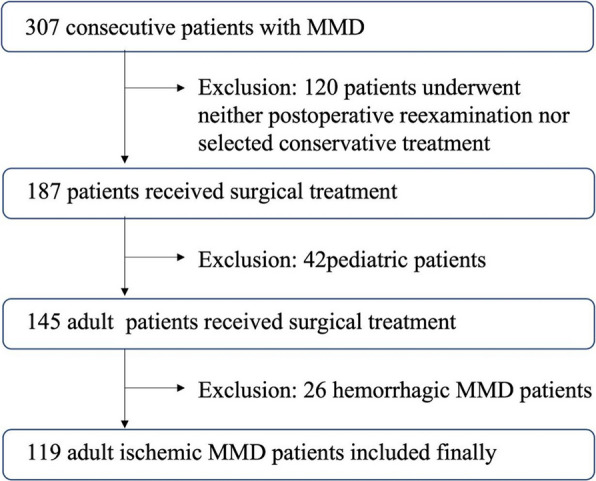
Flow chart of patient inclusion

All cases were from Peking University International Hospital. From June 1, 2021, to October 31, 2024, 307 patients with MMD were admitted to our department. Among them, 120 patients underwent neither a postoperative reexamination nor conservative treatment, and 187 patients received surgical treatment. Forty-two patients were children, and 145 were adults. Among the adult patients, 26 had hemorrhagic MMD, and 119 had ischemic MMD (we considered patients without a previous history of cerebral hemorrhage to have ischemic MMD). We enrolled 119 adult patients who had undergone surgery for ischemic MMD. Coincidentally, all these patients received combined revascularization surgery. A flow chart of patient inclusion is shown in Fig. 1. The diagnosis of MMD was based on the criteria of the Research Committee on Spontaneous Occlusion of the Circle of Willis (2012) [6]. The study was approved by the Ethics Committee of the Peking University International Hospital. Informed consent was obtained from patients for the use of their clinical data.

## Main treatment procedures performed at our center

### Before the operation

No special treatment for hemorrhagic MMD was administered before surgery. Patients with ischemic (non-hemorrhagic) MMD were administered venous fluid replacement therapy 3 days before surgery, and the amount of fluid rehydration was adjusted according to body weight. Low-molecular-weight heparin was administered subcutaneously 3 days before surgery and stopped 12 h before the operation in patients with ischemic MMD to minimize thrombosis.

Patients with cerebral infarction that developed within 1 month or with more than three transient ischemic attacks within 1 month were considered to have contraindications for surgery in our center according to previous studies. These patients were treated with drugs until their symptoms stabilized for at least 1 month, after which revascularization was suggested.

The patient’s blood pressure was monitored four times a day (6:00, 12:00, 18:00, and 22:00) before the operation to obtain basal blood pressure data as a reference. Blood pressure and blood sugar levels must be normal and stable for at least 3 days before surgery.

Cerebral artery DSA (magnetic resonance angiography (MRA) only in children according to the guidelines), head magnetic resonance imaging (MRI), and computerized tomography perfusion (CTP) were completed before surgery.

### Intraoperative procedures

With respect to hemorrhagic MMD, blood pressure was strictly controlled at the basal level, blood pressure was lowered by 10 mmHg after successful bypass, and fluid was administered normally. For ischemia, the anesthesiologist was instructed to administer hypervolemic therapy and to control the patient’s systolic blood pressure to be in the range of 10–20 mmHg above the basal blood pressure without lowering blood pressure after bypass surgery [14].

A computerized tomography (CT) scan was performed 4–6 h after the operation. Head MR images were reexamined 5–7 days after the operation. Head CT scans should be reexamined at any time if the condition changes.

### Surgical methods and key points of the operation

The principles of the surgical strategies are described below. First, the indication for revascularization was based on guidelines established by the Japanese Ministry of Health and Welfare. The symptomatic and hemodynamically affected hemispheres were the preferred sides for revascularization surgery.

Surgical modalities included direct revascularization (end-to-side anastomosis of the branches of the superficial temporal artery to the cortical branches of the middle cerebral artery), indirect revascularization (encephaloduroarteriosynangiosis or EDAS), and combined revascularization. Combined revascularization was first considered for adult patients in our center.

### Image analysis

A newly developed area of low density in the CT scan or a high signal in diffusion weighted imaging (DWI) after the operation was confirmed as a new cerebral infarction.

Cerebral arteriography analysis: Suzuki staging [2] of the bilateral cerebral hemispheres was analyzed for each patient. Lesions characterized by stenosis or occlusion of the major intracranial arteries (ICA, ACA, MCA, and PCA) were measured using handheld digital calipers, and the results were calculated by referring to the WASID criteria [15, 16]. Stenosis was considered present if any segment of a major artery was smaller than its proximal and distal adjacent segments on angiography. The predominantly involved major arteries included the prebifurcation segment of the ICA, the prebifurcation segment of the ACA or the preanterior communicating artery segment, the prebifurcation segment of the MCA, and the prebifurcation segment of the PCA. Assessment of the severity of stenosis in major arteries: A comparison between the minimum diameter at the narrowest segment and the maximum diameter of the distal segment is shown in Fig. 3. We divided lesions in major intracranial arteries into normal, mild stenosis (stenosis < 50%), severe stenosis (stenosis ≥ 50%), and occlusion. With respect to the actual measurement of the degree of vascular stenosis, we found that accurately distinguishing between 50–69% and 70–99% stenosis was difficult. Moreover, in patients with moyamoya disease, a certain major blood vessel is often very slender throughout its entire course. Therefore, we defined “severe stenosis” as any lesion ≥ 50%. All image analyses were performed by 2 neurosurgeons who had more than 10 years of experience in treating patients with MMD and were blinded to the results produced by the other neurosurgeon. A third neurosurgeon who specialized in cerebral vascular disease was consulted when a disagreement occurred.

### Data analysis

Logistic regression analysis was performed to analyze the correlations between the preoperative Suzuki stage, lesions in major intracranial major arteries (ICA, ACA, MCA, and PCA), age, sex, and postoperative infarction. Statistical significance was set at *p* < 0.05. The statistical analysis was performed with IBM SPSS Statistics (version R26.0.0.0).

## Results

**Fig. 2:**
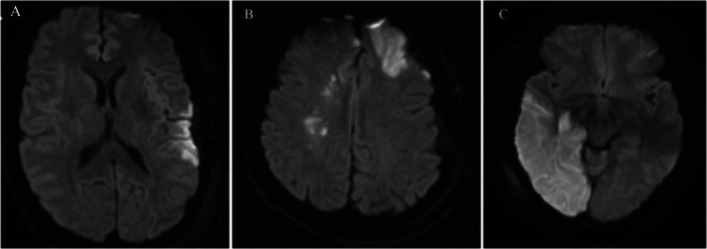
Different types of postoperative infarction. Postoperative DWIs captured using an MRI system. **A** Operative area infarction. **B** Scattered infarction. **C** Major artery infarction

During this period, 307 patients with MMD, including 145 adult patients, were admitted to our center, and 235 patients underwent revascularization operations. After the exclusion of 26 adult patients with hemorrhagic MMD, 119 adult patients with ischemic MMD who underwent a combined revascularization operation were included in the final analysis. When the medical records of patients with postoperative infarction were analyzed together, three distinct types of postoperative infarction were observed; as a categorical analysis is more conducive to identifying possible causes, we classified postoperative infarction as an operative area infarction, where the infarct is strictly confined to the surgical area; scattered infarction, where the infarction sites are discontinuous and present in small patches that are less than the blood supply area of a major artery; and major artery infarction that involves the area supplied by a major artery (ACA, MCA, or PCA) or even the entire area of the ICA (Fig. 2). The first two types of infarction have relatively small areas of damage and a relatively better prognosis. We analyzed only the factors related to the type of infarction in major arteries. New infarctions developed in 11 hemispheres of 8 patients, and 3 patients presented with bilateral infarctions. No ischemic complications occurred in the pediatric patients or patients with hemorrhagic MMD. A significant correlation was not observed between the Suzuki stage and postoperative infarction (odds ratio [OR], 0.815; 95% confidence interval [CI], 0.565–1.178; *p* = 0.277). If the degree of stenosis was considered an ordinal variable, statistically significant correlations were not observed between ICA, ACA, MCA, or PCA lesions and postoperative infarction. When the degree of stenosis was considered a disordered variable and the normal artery was taken as the reference, the logistic regression analysis showed that severe stenosis of the ACA, MCA, and PCA was statistically significantly correlated with postoperative infarction [ACA (OR, 49.500; 95% CI, 5.083–482.011; *p* = 0.001); MCA (OR, 24.000; 95% CI, 1.459–394.881; *p *= 0.026); and PCA (OR, 54.333; 95% CI, 4.307–685.462; *p* = 0.002)] (Table 1).

**Table 1:** Results of the logistic regression analysis

	All	Infarction	No infarction	*p*	OR (95% CI)
Sex				0.753	
Male	53	4 (36.36)	49 (45.37)		
Female	66	7 (63.64)	59 (54.63)		
Suzuki stage				0.277	0.815 (0.565, 1.178)
1	19	1	18		
2	11	1	10		
3	66	4	62		
4	49	3	46		
5	43	1	42		
6	50	1	49		
ICA					
0	167	9	158	1	Reference
1	28	0	27	0.747	0.707 (0.086, 5.820)
2	4	2	2	0.046	8.833 (0.730, 106.821)
3	40	0	40	0.998	0.000 (0.000, 0.000)
ACA					
0	74	9	65	1	Reference
1	15	1	14	0.014	9.429 (1.570, 56.623)
2	3	1	2	0.001	49.500 (5.083, 482.011)
3	146	0	146	0.436	0.455 (0.063, 3.301)
MCA					
0	26	2	24	1	Reference
1	18	4	14	0.999	0.000 (0.000, 0.000)
2	5	3	2	0.026	24.000 (1.459, 394.881)
3	189	2	187	0.981	1.027 (0.123, 8.570)
PCA					
0	168	6	162	1	Reference
1	6	1	5	0.149	5.433 (0.547, 54.000)
2	3	2	1	0.002	54.333 (4.307, 685.462)
3	61	2	59	0.937	0.937 (0.184, 4.772)

The statistical analysis revealed that the type of infarction in the major arteries was significantly associated with severe stenosis of the ACA, MCA, and PCA. Each case of infarction in a major artery was associated with severe stenosis of the feeding artery on preoperative angiography. See the Supplementary information.

Clinically, we immediately performed digital subtraction angiography (DSA) of the cerebral arteries in three patients with severe postoperative dysfunction and excluded patients with intracranial hemorrhage, who were later shown to have severe infarction on CT scans or MRI. On preoperative angiography, arteries with severe stenosis were occluded, or distal blood flow was significantly weakened, which confirmed that the infarction was caused by severe stenosis of the major arteries before the operation. See the case illustration.

### Case illustration

#### Case 1

**Fig. 3:**
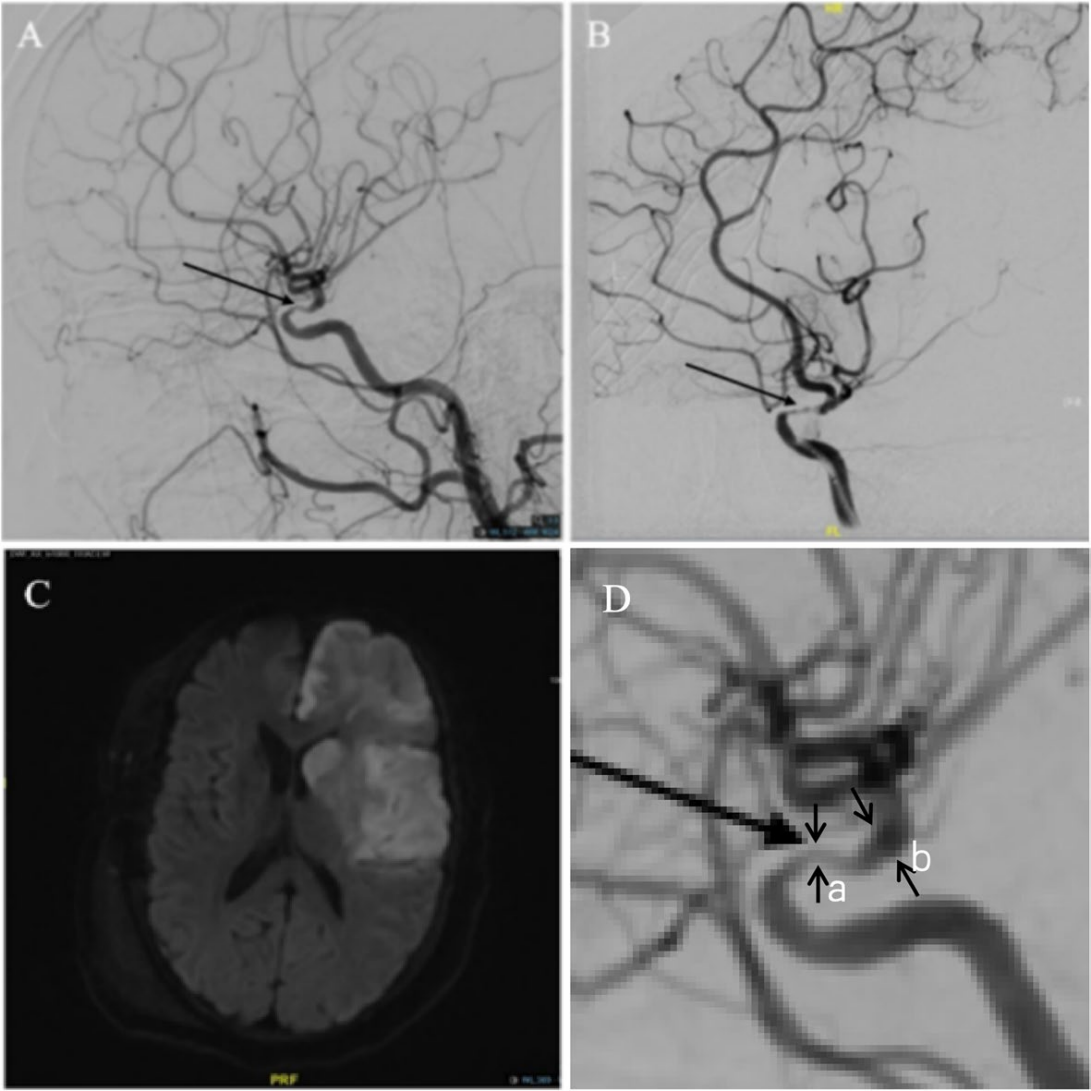
Infarction of a major artery with an aggravation of severe stenosis. DSA and MR images of a 40-year-old female patient with MMD show aggravated stenosis of the terminus of the left ICA (arrow in (**A**), before the operation; arrow in (**B**) after the operation) and new infarction of the ICA area (**C**, diffusion-weighted imaging after recanalization). **D** Magnified view of a part of the image in (**A**), showing the method used to assess the severity of stenosis in major arteries: comparison between the minimum diameter (a) at the narrowest segment and the maximum diameter (b) of the distal segment

A patient with an infarction of a major artery after revascularization (Fig. 3).

A 40-year-old female patient with MMD complained of weakness in the left lower limb; after combined revascularization on the right side, she developed coma, aphasia, and left hemiplegia. DSA was performed immediately, which revealed aggravated stenosis of the terminus of the left ICA. MRI showed a new infarction of the ICA area.

#### Case 2

**Fig. 4:**
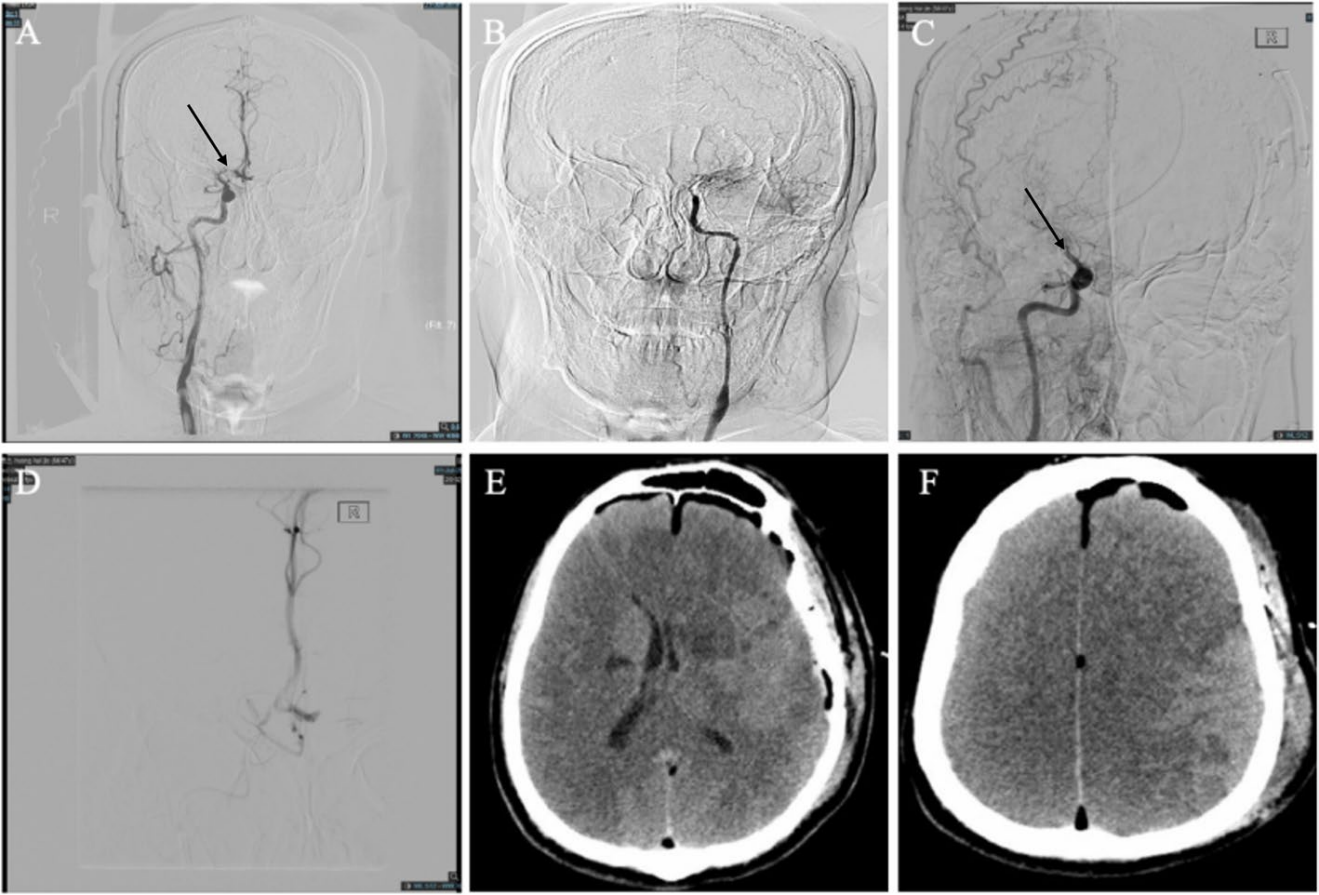
Major artery infarction with severe stenosis and occlusion. DSA and CT images of a 47-year-old male patient with MMD. **A** and (**B**) Right and left ICA angiographs; the arrow in A shows severe stenosis of the first part of the ACA (A1). **C** Right ICA angiograph after revascularization; the arrow shows occlusion of the ACA. **D** Microcatheter angiograph passing through A1 showing that acute occlusion occurred in A1. **E**, **F** CT scan 4 h after the operation showing a newly developed area of low density in the bilateral cerebrum, which is consistent with the A1 supply area

Another patient with an infarction of a major artery after revascularization (Fig. 4).

A 47-year-old male patient complained of bilateral numbness and weakness of the bilateral upper extremities and unresponsiveness. He was still in a coma 2 h after surgery consisting of left-sided combined revascularization. DSA was performed immediately and showed the occlusion of the right internal carotid artery, which had severe stenosis before surgery. A CT scan 4 h later revealed a newly developed low-density region in the bilateral cerebrum.

## Discussion

In our case series, the total incidence of ischemic complications after revascularization was 5.52% (8 of 145 adult patients with MMD, which included 119 patients with the ischemic type and 26 patients with the hemorrhagic type), which is similar to the findings of previous studies in which postoperative ischemic events occurred in 7.6% of adult patients with MMD [7]. The statistical analysis showed that severe stenosis of the major intracranial arteries is significantly associated with infarction of the major arteries after revascularization in adult patients with ischemic MMD. Patients with an infarction in a major artery after surgery, which was later confirmed by DSA, developed the condition due to the acute occlusion or exacerbated stenosis of a major artery that had severe focal stenosis before surgery.

If the degree of stenosis was considered an ordinal variable, no statistically significant correlations were observed between ICA, ACA, MCA, or PCA lesions and postoperative infarction. When the degree of stenosis was considered a disordered variable and the normal artery was taken as the reference, the logistic regression analysis showed that severe stenosis of the ACA, MCA, and PCA was significantly correlated with postoperative infarction.

The results of this analysis suggest that postoperative infarction may not be positively correlated with the severity of stenosis in a major artery. Instead, only severe stenosis (stenosis ≥ 50%) of the major cerebral artery is related to postoperative infarction. This finding is consistent with our clinical experience. However, this issue has not been addressed in previous studies. Moreover, in several cases, DSA revealed acute occlusion of the severely stenotic artery or aggravated stenosis after surgery. This result may be due to the unstable state of a severely stenotic major artery and weakened hemodynamic changes or changes in intracranial pressure, leading to the acute occlusion of the severely stenotic arteries [17, 18]. At this time, the blood supply to the distal segment of the severely stenotic artery depends on the narrow artery cavity, and compensatory vessels have not yet formed. Once the arterial cavity is occluded or aggravated, the blood supply area of the major artery is completely infarcted. However, whether the acute occlusion is due to thrombosis or acute constriction of the vessel cannot be determined. Previous studies have shown that the higher the Suzuki stage is, the higher the risk of postoperative infarction [7]. However, this phenomenon was not observed in our data. Our analysis showed that mild stenosis or occlusion of the major intracranial arteries was not associated with postoperative infarction. Suzuki stages were defined according to the lesions in the major arteries, from mild stenosis to occlusion [2]. However, our study revealed that the risk of postoperative infarction did not increase with the severity of stenosis in the major arteries. Only when the degree of stenosis was sufficiently severe but not occluded or mild was postoperative infarction prone to occur, which could explain why the statistical analysis showed no association between the Suzuki stage and postoperative infarction. Moreover, the Suzuki stage serves as a global indicator of disease progression—encompassing the development of the collateral circulation—and thus may not directly reflect the focal hemodynamic instability of individual major arteries. However, the extremely wide confidence intervals suggest statistical instability and possible overfitting. This result could be attributed to the insufficient sample size of infarction cases. We reexamined the four cases of postoperative infarction using DSA to compensate for the small number of cases and identify more precise causes. Angiography confirmed that the major artery with severe stenosis preoperatively was occluded. This finding was consistent with the area of the new cerebral infarction and suggested that the occluded vessel was the vessel responsible for the acute infarction after the operation.

Similar to atherosclerotic stenosis, a severely stenotic major artery is prone to acute occlusion [16]. Studies concerning atherosclerotic intracranial arterial stenosis have shown that the degree of stenosis and collateral circulation affect the risk of recurrent ischemic events and outcomes. Robust antegrade flow of the major intracranial artery and good collaterals were protective against stroke. The WASID study revealed that stenosis of a major intracranial artery with a percentage of stenosis greater than 70% was associated with an increased risk of recurrent stroke compared with that in patients with less stenosis [16]. Severe stenosis of the major intracranial arteries in MMD patients increases infarction complications after revascularization surgery, which may be similar to the increase in the incidence of recurrent stroke observed in patients with atherosclerotic intracranial arterial stenosis. Hypoperfusion, artery-to-artery embolism, and plaque extension over the small penetrating artery ostia (also known as branch atheromatous disease) are three main hypothesized mechanisms of stroke related to atherosclerotic intracranial arterial stenosis. According to our case series illustrated above and the pathological features of MMD, the mechanisms underlying acute ischemic events in MMD patients after surgery might also include acute retraction of the severely stenotic artery. In addition to the aforementioned types of infarction in major arteries, scattered infarctions and operative area infarctions were present but did not receive our attention.

### Limitations

First, this retrospective study has a natural limitation, as it can identify only some related risk factors. Second, as mentioned in the Background section, we focused mainly on the relationships between lesions in intracranial major arteries and postoperative cerebral infarction and gave little consideration to other confounding factors, such as age and the side of the operation. Third, this study was conducted at a single center, and patient enrollment and the surgical technique used might be sources of bias; the small number of infarction events limited the statistical power.

## Conclusions

Severe stenosis of major intracranial arteries is an important risk factor for an infarction of a major artery after combined extracranial–intracranial revascularization in adult patients with ischemic MMD.

## Supplementary Information

Supplementary Material 1. Figure S1. Major artery infarctions 3. Preoperative DSA shows severe stenosis of terminal of right ICA, newly developed infarction of right ICA feeding area after revascularization. Figure S2. Major artery infarctions 4, Preoperative DSA shows severe stenosis of first part of left ACA and MCA, newly developed infarction of left ACA and MCA feeding area after revascularization. Figure S3. Preoperative DSA shows severe stenosis of first part of right PCAand non-embryonal posterior cerebral artery, newly developed infarction of the right PCA feeding area after revascularization.

## Data Availability

The datasets used and/or analyzed during the current study are available from the corresponding author upon reasonable request.

## Abbreviations

ACA: Anterior cerebral artery
CI: Confidence interval
CO2: Carbon dioxide
CT: Computed tomography
CTP: Computed tomography perfusion
DSA: Digital subtraction angiography
DWI: Diffusion-weighted imaging
EDAS: Encephaloduroarteriosynangiosis
ICA: Internal carotid artery
OR: Odds ratio
PCA: Posterior cerebral artery
MCA: Middle cerebral artery
MMD: Moyamoya disease
MRA: Magnetic resonance angiography
MRI: Magnetic resonance imaging
