# Supplementary material for: Severe stenosis of major intracranial arteries: an important risk factor for infarction complications after combined revascularization in adult patients with ischemic moyamoya disease
Source: Chin Neurosurg J. 2026 Apr 14;12:12. doi: 10.1186/s41016-026-00430-0 (PMC13078013; doi:10.1186/s41016-026-00430-0)
Supplement: Supplementary file 1 — Supplementary Material 1. Figure S1. Major artery infarctions 3. Preoperative DSA shows severe stenosis of terminal of right ICA, newly developed infarction of right ICA feeding area after revascularization. Figure S2. Major artery infarctions 4, Preoperative DSA shows severe stenosis of first part of left ACA and MCA, newly developed infarction of left ACA and MCA feeding area after revascularization. Figure S3. Preoperative DSA shows severe stenosis of first part of right PCAand non-embryonal posterior cerebral artery, newly developed infarction of the right PCA feeding area after revascularization. [file 41016_2026_430_MOESM1_ESM.docx]

**Supplemental material**

Figure 1. major artery infarctions 3


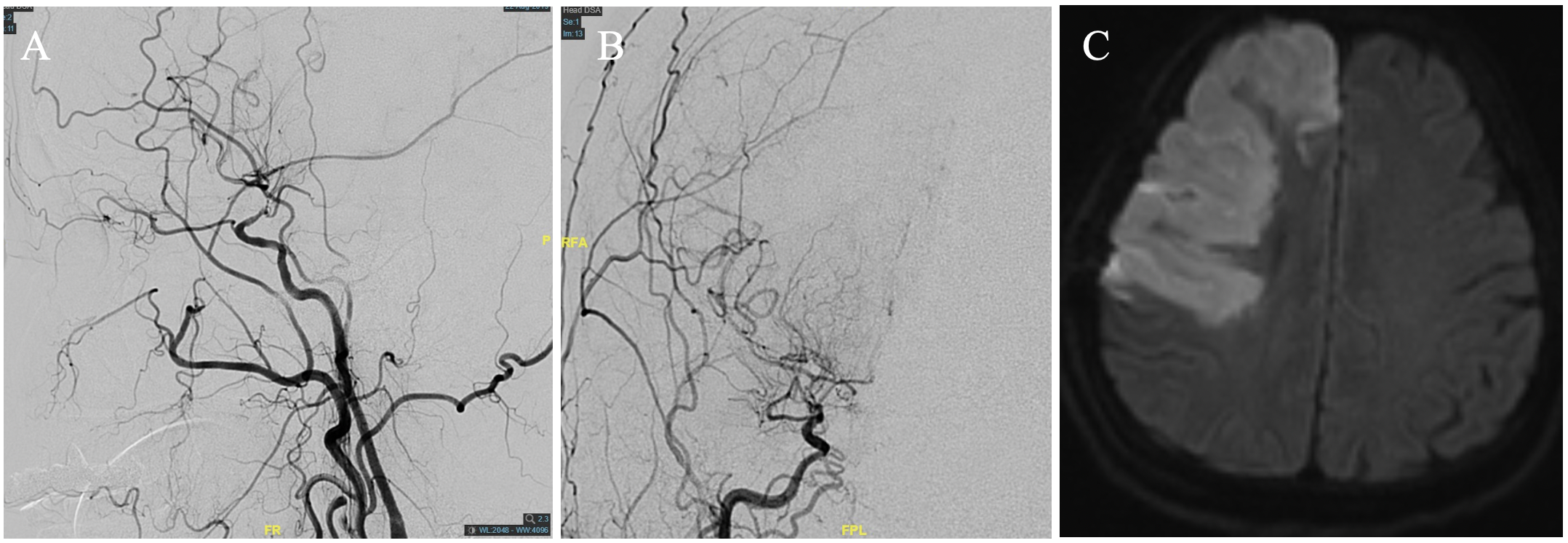


Preoperative DSA shows severe stenosis of terminal of right ICA(A and B), newly developed infarction of right ICA feeding area after revascularization.

Figure 2. major artery infarctions 4


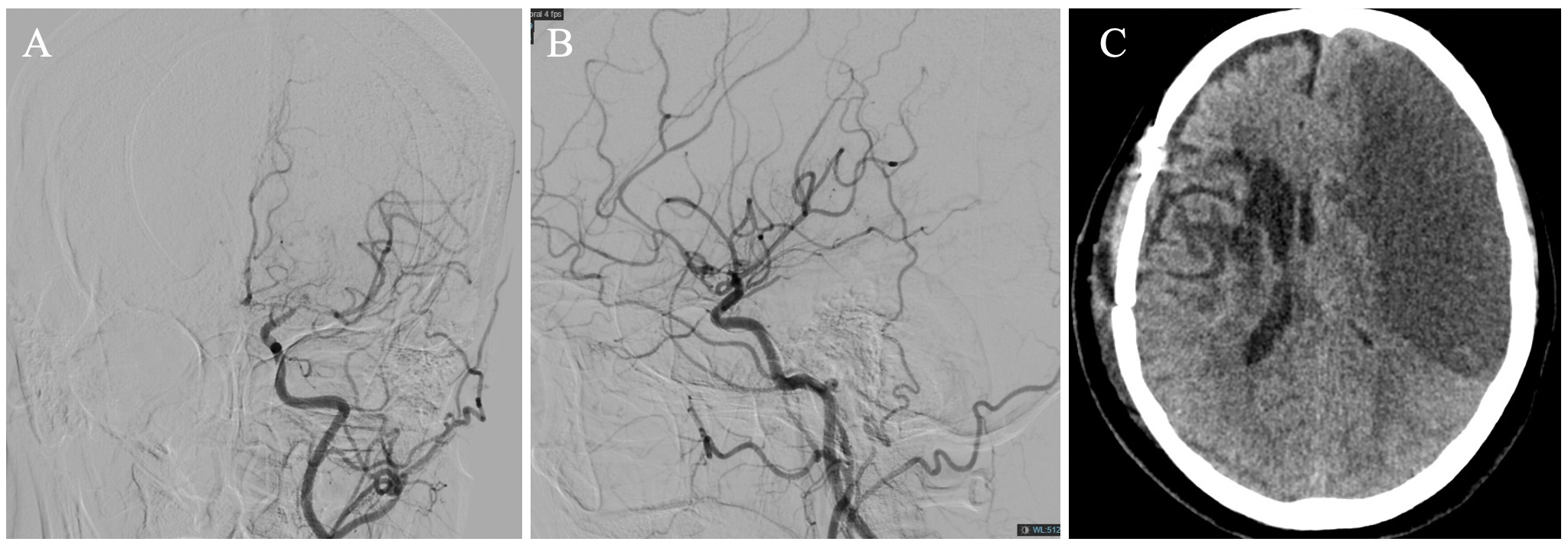


Preoperative DSA shows severe stenosis of first part of left ACA and MCA(A and B), newly developed infarction of left ACA and MCA feeding area after revascularization.

Figure 3


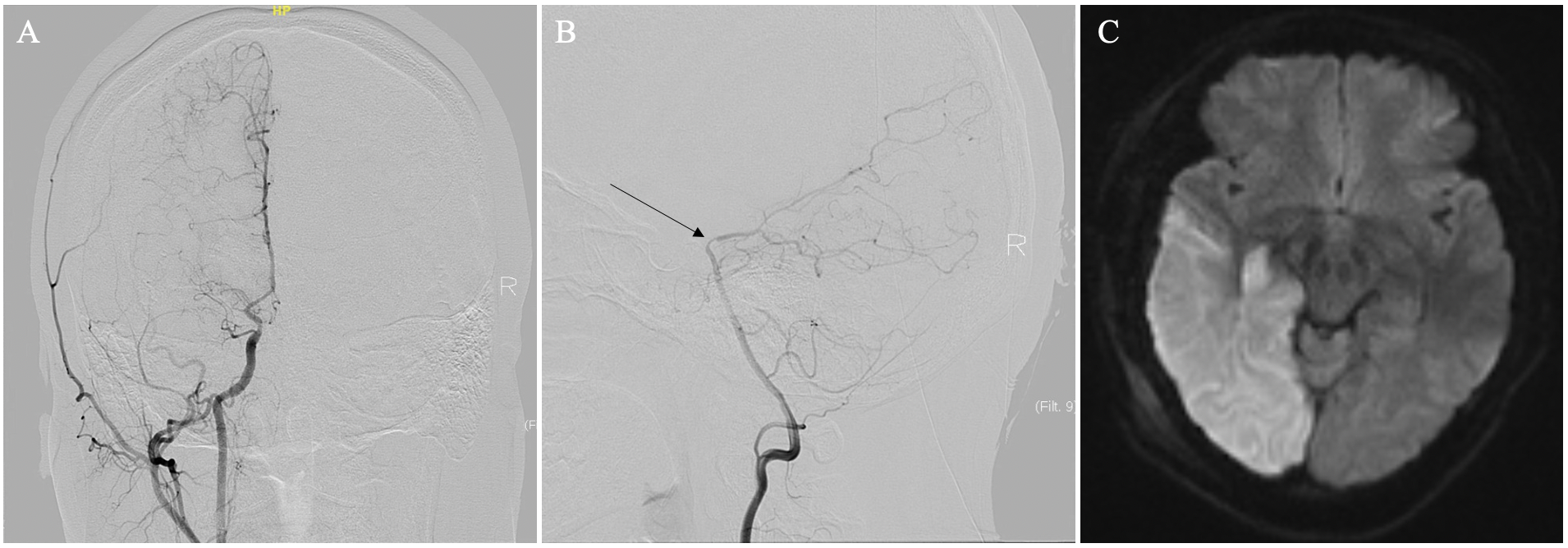


Preoperative DSA shows severe stenosis of first part of right PCA(B, arrow) and non-embryonal posterior cerebral artery(A), newly developed infarction of the right PCA feeding area after revascularization.
